# Costing Human Rights and Community Support Interventions as a Part of Universal Access to HIV Treatment and Care in a Southern African Setting

**DOI:** 10.2174/157016211798038614

**Published:** 2011-09

**Authors:** Louisa Jones, Paula Akugizibwe, Michaela Clayton, Joseph J Amon, Miriam Lewis Sabin, Rod Bennett, Christine Stegling, Rachel Baggaley, James G Kahn, Charles B Holmes, Navneet Garg, Carla Makhlouf Obermeyer, Christina DeFilippo Mack, Phoebe Williams, Caoimhe Smyth, Marco Vitoria, Siobhan Crowley, Brian Williams, Craig McClure, Reuben Granich, Gottfried Hirnschall

**Affiliations:** 1 World Health Organization, Geneva, Switzerland;; 2 AIDS and Rights Alliance for Southern Africa (ARASA), Namibia;; 3 Human Rights Watch, New York, USA;; 4 Independent Consultant, UK;; 5 AIDS Alliance, Hove, UK;; 6 University of California, San Francisco, USA;; 7 Office of the US Global AIDS Coordinator, Department of State, Washington DC, USA;; 8 Vestergaard-Frandsen, Lausanne, Switzerland;; 9 University of North Carolina, Chapel Hill, North Carolina, USA;; 10 South African Centre for Epidemiological Modelling and Analysis, Stellenbosch, South Africa

**Keywords:** Cost analysis, economics, HAART, highly active antiretroviral therapy, HIV prevention, human rights.

## Abstract

Expanding access to antiretroviral therapy (ART) has both individual health benefits and potential to decrease HIV incidence. Ensuring access to HIV services is a significant human rights issue and successful programmes require adequate human rights protections and community support. However, the cost of specific human rights and community support interventions for equitable, sustainable and non-discriminatory access to ART are not well described. Human rights and community support interventions were identified using the literature and through consultations with experts. Specific costs were then determined for these health sector interventions. Population and epidemic data were provided through the Statistics South Africa 2009 national mid-year estimates. Costs of scale up of HIV prevention and treatment were taken from recently published estimates. Interventions addressed access to services, minimising stigma and discrimination against people living with HIV, confidentiality, informed consent and counselling quality. Integrated HIV programme interventions included training for counsellors, *‘Know Your Rights’* information desks, outreach campaigns for most at risk populations, and adherence support. Complementary measures included post-service interviews, human rights abuse monitoring, transportation costs, legal assistance, and funding for human rights and community support organisations. Other essential non-health sector interventions were identified but not included in the costing framework. The annual costs for the human rights and community support interventions are United States (US) $63.8 million (US $1.22 *per capita*), representing 1.5% of total health sector HIV programme costs. Respect for human rights and community engagement can be understood both as an obligation of expanded ART programmes and as a critically important factor in their success. Basic rights-based and community support interventions constitute only a small percentage of overall programmes costs. ART programs should consider measuring the cost and impact of human rights and community support interventions as key aspects of successful programme expansion.

## INTRODUCTION

In 2010, an estimated 34 million people were living with HIV and the majority of people with HIV were living in low- and middle-income countries [1,2,3]. This disproportionate HIV burden is often compounded by the lack of quality prevention, testing and treatment services. After the G8 commitment in 2005, all United Nations member states pledged to achieve universal access to quality HIV prevention, testing and treatment by 2010 [4, 5]. This builds on the 2003 *‘3 by 5’* initiative, the broader Millennium Development Goals (MDGs) [6] and the 2010 push for treatment optimisation through the World Health Organization (WHO)/Joint United Nations Programme on HIV/AIDS (UNAIDS) *Treatment 2.0* platform [7]. These efforts reflect the international community’s commitment to combat HIV/AIDS and recognise human rights as “an essential element in the global response to the HIV/AIDS pandemic” [8].

UNAIDS estimated that by the end of 2010 6.6 million people were taking antiretroviral therapy (ART), around 42% of those in need as defined by the 2010 WHO recommendations for ART for those with CD4 cell count ≤ 350 cells/mm^3^ [1,2,3]. Despite this achievement, around 9 million people were estimated to be eligible and still in need of treatment in 2010, with substantial patient attrition along the treatment cascade from HIV testing and staging to ART initiation and continuation [1,3,9,10,11]. Furthermore, only around 40% of people living with HIV know their status and the number of new infections continues to add to the future caseload, with around 2.6 million new infections estimated in 2010 alone [1, 3, 12]. Moreover, virtually everyone with HIV will eventually need ART to survive so as many as 28 million people are waiting, mostly without knowing that they are living with HIV, to become ART-eligible before they sicken or die [1, 2].

The concept of expanding access to ART is integral to the human right to health and provides a significant benefit for individuals and the community. However, in some settings, human rights abuses have been reported in association with HIV testing and counselling and treatment programmes [14-16]. It is essential to improve and expand approaches which increase access to HIV testing and treatment while ensuring respect for and protection of human rights. This is not only an ethical obligation of HIV programmes, but also critical to increased uptake of HIV testing, counselling and treatment, reducing vulnerability to infection and ensuring the success of ART expansion.

The basic human right to health care including HIV services remains a major human rights shortfall in most parts of the world. Human rights advocacy has emphasised how human rights abuses increase vulnerability to HIV infection and impede effective HIV responses [8, 17]. Other HIV-related human rights abuses have been widely reported, including those related to access to accurate and comprehensive HIV prevention and treatment information [18,19], HIV testing [16], equitable access to treatment for criminalised populations and prisoners [20-24], men who have sex with men [25], migrants [26, 27], women [28-30], and children [31-33]. People living with HIV have been subject to violations of human rights on the basis of their HIV status, including lack of access to care, experiencing stigma and discrimination, loss of property, employment, freedom and health. There is a clear intersection between the right to health and the realisation of other human rights. For example, gender inequality and entrenched discrimination on the basis of sexual orientation not only undercut mental and physical health status, but also hinder access to prevention and treatment programmes, thus increasing individual and community vulnerability to HIV. There are increasing calls for human rights and community-support specific planning and funding as an integral element of efforts to reach universal access to high quality HIV services [34]. Despite this growing recognition, these interventions are rarely costed or included in HIV programming to improve access and health outcomes.

Ensuring access to high quality ART requires community engagement within a strong human rights framework. To examine the potential costs of integrating human rights and community support into routine programming, we constructed a detailed hypothetical costing framework using South Africa’s health infrastructure [35]. We considered both integrated and complementary health sector interventions. Although we identified them, we did not include non-health sector costs in the costing framework (Table **1**). Ultimately, a successful response to the HIV epidemic would also require non-health sector human rights interventions to address broader socio-economic issues beyond the narrow health sector.

## METHODS FOR BUILDING THE COSTING FRAMEWORK

We used a standard search strategy of the published literature to determine the human rights and community support interventions for the costing framework. We consulted experts and key informants regarding published and unpublished sources of information. The costing framework of the ART programme and the comparison of relative costs of the potential human rights and community support interventions is based on South African and other sub-Saharan data [34] and recently published HIV programme cost models [36]. We applied the 80% treatment target from the *South African National Strategic Plan for HIV and AIDS* and the WHO recommended eligibility criteria of CD4 ≤350 cells/mm^3^. In South Africa, the projected costs for CD4≤200 is United States (US)$58.45 *per capita* in 2015 after rapid scale up to 80% coverage, compared to US$32.32 in 2008 [37]. We used the WHO methodology [2] to estimate the proportions eligible for treatment, which for CD4≤350 would translate into a 43% increase to US $4375 billion over the projected cost for the CD4≤200 criteria used for the 2015 programme projections [36, 38]. Demographic data are taken from government publications [38] and we projected the population growth at 1% *per annum* in line with the historic trend and the published projection of 1.5% *per annum* annual growth in HIV prevalence to 2015 [36].

To reach the 80% treatment target, we included provider-initiated HIV testing and counselling (PITC) [39], targeted interventions for marginalised groups, and an annual community-based campaign in 2909 sites in 9 provinces [40]. Costing of the expansion of access to HIV testing and counselling included 20% in health care facilities and 80% through community-based campaigns. PITC costs were derived from routine programme costs and the community campaign costs are based on the detailed costing of a 2008 private-public health campaign in Kenya for which extensive and detailed data were available [40, 41]. Kenyan campaign costs were adjusted for South Africa in accordance with International Monetary Fund (IMF) purchasing power parity ratios to align with US dollar treatment costs.

**Fig. (1):**
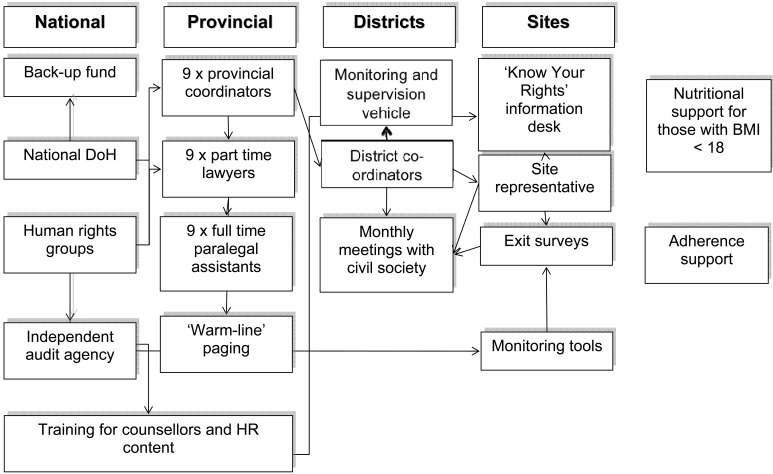
**Schematic diagram showing the human rights measures included in costing according to level of intervention (national, provincial, district, site, community).** DoH, Department of Health; HR, human rights; BMI, body mass index.

Some of the health sector human rights and community support interventions were costed as integrated into routine HIV services programme scale-up. We also costed additional complementary health sector human rights and community support interventions that are not routinely considered as part of HIV services. Although non-health sector human rights interventions, such as the provision of shelter for domestic violence survivors, law reform, and other efforts are at the core of human rights programming, they were considered beyond the scope of this costing exercise which was limited to the health sector. A summary diagram demonstrates the components of the human rights and community support elements included in the costing framework (Fig. **1**).

## HUMAN RIGHTS AND COMMUNITY SUPPORT INTERVENTIONS

**Fig. (2):**
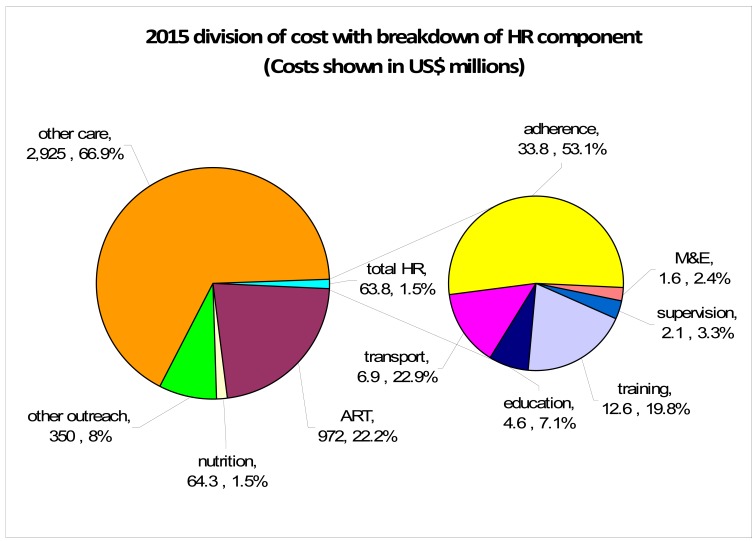
Estimated annual HIV programme costs and percentages by category with breakdown of human rights and community support components (2015). Care includes pre ART and on ART costs for hospitalization, primary health care, and laboratory. ART includes the cost of 1st and 2nd line. Nutrition is provided to those on ART with BMI<18. Outreach includes campaign- and facility-based HIV testing and counselling costs. Nutrition is included in wider programme but not human rights and community support costs. Total human rights costs are 1.5% (US$63.8 million) of the annual HIV programme. M&E is monitoring and evaluation.

Human rights and community support issues, measures to address these issues and their associated costs are discussed below. In Table **2**, specific interventions and the associated costs are displayed for each issue identified, including the levels at which the intervention is provided (national, provincial, district, community; see Fig. **1**). Figures showing costing breakdown are also included (Fig. **2**).

### Improved Access to Health Care

Adequate access to health care is an important aspect of the basic human right to health [42]. In resource-constrained settings, lack of access negatively impacts the control of most preventable disease and our ability to improve access will likely make the difference between achieving universal access and the MDGs [43]. A recent systematic review identified the barriers to provision of ART in resource-limited settings [44], dividing obstacles into economic, sociocultural and behavioural. Other reviews have identified nutrition, community support, as well as legal and political barriers including human rights abuses, as serious obstacles.

Economic barriers identified within the scope of the costing framework included transportation costs to HIV testing and counselling and treatment services [29, 33, 45], perceived costs of treatment, testing and counselling [29, 45-50], and job loss due to HIV positive diagnosis [28, 49]. User fees are another common barrier linked to poor adherence but are not included in the costing framework since we considered public sector service delivery which does not include user fees [29, 33, 37]. Marginalised groups are particularly vulnerable to HIV and we costed interventions to reduce stigma and mobilise these communities for improved access to services. In resource-poor settings, people living with HIV may not be able to obtain sufficient nutrition or afford travel to seek services [33, 44]. In the absence of home-based care or other outreach, people are unlikely to seek medical care if costs involved mean forgoing food for the sake of travel costs. We included the cost of buses and minibuses for transportation to health services for more distant parts of the community as well as accessible transport for disabled individuals (US $6.9 million per year) and nutritional support for those with a body mass index (BMI) less than 18 in order to improve health outcomes (US $333 per person treated, US $64.3 million in South Africa).

Additional support at the community level to promote adherence to ART has been shown to be effective in some settings [51]. Although addressing adherence is complex and there may be multiple approaches, potential adherence support currently exists in South Africa in the form of tuberculosis (TB) directly observed therapy (DOT) workers. DOTS workers would require re-orientation to provide adherence support for people on ART (not DOT) and would provide an important link between HIV and TB services. Table **3** illustrates selected activities that could be provided by community support and human rights workers. In some settings, around 70% of incident TB cases have HIV [52] and we assumed that there will be a 5% overlap in the numbers of community support workers needed. Overall TB DOTS workers will provide a small but important level of support (5%) for those with HIV associated TB, therefore, we costed one community support worker per 120 individuals on ART (US $1440 per worker per annum) for the remaining 95% of those on treatment. In addition to this, one community support supervisor per community site (2909 sites) will be provided (US $2667 per community per annum).

Sociocultural barriers to accessing HIV services identified were HIV/AIDS-related stigma and discrimination [29-31, 33, 45, 50, 53], gender inequalities [30, 54], cultural and social norms leading to marginalisation of certain minority groups [20, 25, 44], and those related to health care system deficiencies, including poorly trained health care providers [45, 46, 54, 55]. Relevant behavioural issues were personal denial of illness [46, 49, 56], perception of medical testing and care as expensive [45, 46, 48-50], and lack of education or awareness of treatment or diagnosis options and availability [19, 33, 45, 46, 49, 54, 57-59]. In addition, personal factors included refusal to seek medical care unless very ill [46, 47, 50], denial of being at risk of infection, inability to attend testing due to work or family responsibilities, mental health issues [60] and fear of medical providers [46]. Although health care worker training and communications materials will include discussion of these and other issues, specific interventions to address these barriers are not costed (see Table **1** for selected non-health sector interventions). Expanding access to HIV services within a human rights framework may address some of the sociocultural barriers as health care workers and the community recognize the increasing numbers of people accessing HIV testing and counselling and ART.

Punitive laws, policies and regulations are common barriers to accessing health care worldwide [61], particularly for marginalised or criminalised populations [62, 63]. According to the 2008 UNAIDS Global Report, 63% of countries that reported have laws, regulations, or policies that impede access to HIV prevention, treatment, care and support among populations most at risk of HIV infection. In many parts of the world, legislation effectively criminalises populations most at risk of HIV infection, such as sex workers, drug users, and men who have sex with men. This fuels stigma and discrimination, increases barriers to HIV information and treatment, and contributes to the spread of the disease. Laws criminalising HIV transmission can discourage HIV testing, potentially subjecting those who know their HIV status to criminal penalties while exempting those who are unaware of their infection [64-67]. Since 2005, 14 countries in Africa have passed HIV-specific laws that potentially criminalise all sexual behaviour among HIV-positive individuals, including those who use condoms, regardless of disclosure and actual risk of transmission. More than 80 countries have legislation that prohibits same-sex behaviour [68]. In a number of countries, maternal-to-child HIV transmission is a criminal offense, even where ART may not be available.

Another common issue is work place discrimination, resulting in loss of employment, or pre-employment testing which prevents people from getting employment in the first place. For many people living with HIV, this is the most common human rights violation. Although not costed, law reform is necessary and will involve capacity building of law and policy makers to understand the linkages between rights and health, and for the need to remove punitive laws (Table **1**). While recognising the need to address the larger legal and social issues, we costed legal assistance in the form of a part-time lawyer (0.3 full-time equivalent at US $7524 *per annum*) and a full-time paralegal assistant for each province (US $12,319). Selection and contracting of the legal support would be managed by independent human rights and community support organisations. Legal assistance could be accessed by a "warm line" paging system, with the existence of a one-time back-up fund of US $100,000 for longer-term legal representation. We also costed community-based “*Know Your Rights*” campaigns to help build the capacity of communities to claim rights and advocate for judicial reform including the removal of punitive laws.

### Consent, Counselling and Confidentiality

Informed consent and confidentiality of HIV results are cornerstones of HIV testing and counselling and clinical services [69, 70]. Individuals should be confident that their rights will be respected when accessing HIV testing and counselling, including their right to decline testing without suffering negative consequences, such as the denial of other health care [23]. To encourage access to testing while respecting rights, it is important that HIV testing is available in different settings, and individuals should be able to seek testing where they feel most confident that their rights will be respected. The framework includes both facility- and community-based testing options with the majority of testing taking place in the community. PITC is designed to improve access to knowing one’s HIV status and is intended to be ‘opt-out’ (requiring patients to decline routine HIV testing) [71-74]. To respect patients’ rights, HIV testing and counselling, needs to be both readily available and voluntary without coercion and with a fully informed process including awareness of the right to refuse. Although most facility-based and community-based HIV testing is conducted without incident, patients may feel unable to refuse a test when requested by the health care provider [71-74]. Concerns have been raised that provider-initiated testing approaches may dissuade people from seeking medical care [74, 75]; however, PITC programs have helped to improve access to HIV testing and counselling for millions of people [76, 77]. ‘Normalising’ HIV testing and counselling, including couples counselling, and integrating it with other services and community campaign-based approaches may assist in minimising the impact of stigma and discrimination [78,40]. Improved access to ART, particularly home-based ART care, may also lead to a reduction in stigma experienced by HIV-positive individuals [79-84]. Health care worker education is included in routine training and education for all counsellors (campaign- and facility-based testing and counselling) and is currently costed at US $12.6 million *per annum*.

Regardless of the testing venue, obtaining informed consent requires that the individual is sufficiently informed on a subject to agree to a procedure [69, 70]. In our costing framework, information for most clients will be provided prior to actual testing by individual briefings by counsellors and campaign-based education. People who test positive or negative will also require information regarding treatment options, support and other interventions to maintain HIV negative status [69, 70]. We included resources for human rights and community support organisations to monitor HIV testing and counselling and work with health care providers and patient groups to develop educational materials (cost US $261,425).

Confidentiality of testing, results and HIV status should be considered as part of routine programme expansion. In small communities this may be difficult, with surveys of voluntary counselling and testing (VCT) clients demonstrating that potential breaches of confidentiality are a significant deterrent for uptake of testing [85]. Providing different venues for testing and counselling including PITC and community-based settings is one potential approach to decreasing this potential deterrent. For those that test positive, the difficulty of maintaining confidentiality while accessing continuing care is also a concern. Costing included dissemination of information regarding the facility-based and campaign process and the establishment of multiple testing sites. The inclusion of youth in community-based HIV testing campaigns also requires specific attention [86]. In addition to standard training for counsellors, the costing includes an additional day of human rights-focussed training with a stigma reduction intervention for counsellors (US $9.47 million *per annum*) [87]. Costing included support for human rights and community organizations to provide input on training and materials relevant to the training of counsellors. The *'Know Your Rights Desk,'* where participants can gather information about human rights and support services, includes private counselling space for dealing with specific cases in a sensitive and confidential manner (US $231.60 per site, US $673,737 *per annum* for 2909 sites).

### Monitoring and Evaluation

High quality programmes that include a human rights-based approach are based on accountability [61]. UNAIDS and WHO recommend that programmes use monitoring and evaluation (M&E) systems to provide timely feedback on implementation progress and quality of services [88]. Dedicated human rights staff (site-specific human rights and community support representative, district supervisors and provincial coordinators) as well as human rights bodies operating at a national level, need to be engaged in integrated M&E activities as well as periodic surveys. The measures suggested in this paper are intended to improve the human rights framework for successful implementation of ART programmess. However, the actual impact of these human rights interventions are unknown, and the impact in improving quality of services and achieving public health and individual health objectives should be measured. In the costing framework, district supervisors will be responsible for ensuring that the facility HIV testing and counselling and community-based campaign are well-implemented from a human rights and community-support perspective. Monthly meetings between civil society organisations and the district supervisor have also been costed. The framework includes support for a human rights supervisor for each province (US $12,373 per supervisor or US $111,353 *per annum* for 9 provinces). District supervisors are costed at US $655,743 plus US $795,000 for vehicles, while monthly meetings will cost US $38,400 per province, US $350,600 *per annum*. Feedback on the services are costed for through exit surveys, counsellors, district and site staff, which will require collation by an independent M&E contractor and timely action on feedback. These potential activities have been costed at US $150,000 *per annum* for the M&E contractor, and a further US $673,737 *per annum* for the exit surveys from the 2909 sites.

### Stigma and Discrimination

Although in some settings conditions have improved, it is widely recognised that people living with HIV are subject to significant stigma and discrimination [89, 90, 91]. Stigma and discrimination in turn may lead to reluctance to be tested, barriers in seeking testing or health care [44], limited uptake of preventive behaviours [92], social isolation and ostracism [93], harassment, discriminatory behaviours in relation to employment, health care, insurance, ownership of property and education, physical violence and anger from others, including family members [94]. Human rights and community support organisations are costed to adapt and develop new monitoring and training tools, including stigma scales for people living with HIV, [95, 96] to help health care workers in the identification of individuals experiencing stigma while accessing expanded HIV services (US $6,361 per province, US $57,248 *per annum*).

### Special Needs of Women Living with HIV

Women constitute the majority of those infected with HIV in sub-Saharan Africa [97] and are often at increased risk of becoming HIV infected due to the inability to enforce safer sex decisions [98, 99, 100]. This vulnerability is often a result of skewed power relations within the relationship which can include an inability to negotiate safer sex or leave due to economic dependence [101]. Poverty, disruption by civil war, spousal death due to HIV, and lack of protective policy, laws and enforcement can lead to women being forced to resort to survival and transactional sex. Women are often more likely to have negative experiences as the result of a positive HIV test, including blame for infection, abuse or violence from their partner, exclusion from family or home and loss of property [29, 30, 102-106]. In anticipation of these outcomes, women may refuse testing [107-109]. Women may also feel unable to consent for testing without explicit consent, or may be prevented from testing by their partner [110]. This outlines the need for policy change to protect the rights of women to equality, property ownership, freedom from abuse and sexual violence, and to realisation of health. In integrating a human rights- and community-based approach, expanding access to human rights and community support interventions within the health sector response could improve access to life-saving HIV services for women.

For HIV-positive individuals suffering abuse at the hands of an intimate partner, the need for support measures including helplines has also been identified [30, 74, 94, 102, 104, 111]. Support is costed for skills training and protocols for counsellors to identify and counsel women who feel at risk or have experienced inter-personal violence. Campaign and PITC informational materials are intended to educate women about domestic violence and inform them of available resources, help-lines in South Africa and legal rights. Human rights and community support organisations are supported within the framework to work with health authorities to develop relevant human rights tools and protocols (see above). Access to a telephone or other communications is assumed to be available at a community centre, primary health or HIV treatment facility.

Couples and partners counselling has been used as a successful prevention intervention [112, 113-115] and a criticism of previous testing campaigns has been the lack of emphasis on couples-specific counselling [16]. Studies have demonstrated the potential of this type of counselling in terms of prevention of HIV infection by facilitating status disclosure to partners [116, 117]. Couples counselling, by providing a safe environment for disclosure, has the potential to assist in reducing incidences of domestic violence in serodiscordant couples [117]. Pre-campaign social mobilisation will stress the benefits of couples testing, and the campaign will support couples testing. Costing included specific training for counsellors to be better equipped to address potential spousal refusal, to assist disclosure of status to partners, and to counsel serodiscordant couples (included in the overall counsellor training cost of US $9.47 million above) [117].

As part of a multi-faceted societal response, law enforcement agencies must be trained to deal with violence directed against women, HIV-positive individuals and members of most at risk populations. This matter is out of the scope of the costing framework; however, it is anticipated that human rights and community support organisations will provide advice on the appropriate training and materials.

Shelters for women have been recognised as a scarce resource for which there is great need [118]. Few are offered in South Africa, and even fewer have the capacity to care for women who are ill [119]. The need for short-term shelters for women living with HIV is critical, both for those who require medical care and/or those who have suffered domestic violence. Although the best outcome is often a resolution in which a woman can remain in her community with her children, there is also a need for long-term solutions for women who can no longer return home. Women’s shelters are beyond the scope of health sector funding/resources and therefore are not directly costed in the model. Collaboration with organisations that advocate against violence against women and provide support would be needed to successfully implement various components of this model.

### Special Needs of Pregnant Women

Women who are pregnant face additional barriers. For women who test HIV positive, ART is recommended for those eligible and antiretrovirals are recommended for others to prevent mother-to-child transmission (MTCT) [120]. Although potentially life-saving for the mother, the unborn child and partners, these interventions increase the likelihood of disclosure of a woman's positive HIV status and therefore may expose a mother to stigma and discrimination. Support services and education are needed for HIV-positive pregnant women including follow-up with prevention of MTCT programmes, and these would be offered as part of post-test counselling [120]. This is currently costed in the framework as part of the counselling.

### Special Needs of Most at Risk Populations

Although in a generalised epidemic setting people who are sexually active are often most at risk, traditional most at risk populations have been more narrowly defined and include male and female sex workers, men who have sex with men, people who inject drugs, and prisoners. In addition to frequently being criminalised and subject to police violence [121], it has been reported that only 10% of funding for prevention strategies in many countries is allocated to key populations [97]. Unless these populations have access to legal services, human rights protections including law reform and community support services, and funding specifically allocated to working with them, it is possible that the coverage of the expanded ART programme will be poor. Additionally, legal barriers to accessing ART, fragile social support networks, transactional partnerships, and high levels of stigma impede access to services and may also make these populations less likely to disclose their HIV-positive status to partners [83, 122]. Support is costed to access most at risk populations through services in 291 locations designed and led by community leaders. Costing includes counsellor training in issues regarding the needs of most at risk populations as part of human rights training day (included as part of the US $9.47 million for training for the broader community-based outreach).

### Bottom Line: Projecting the Health Sector Costs

The public sector costs of expanded access to ART including human rights and community support is illustrated in Fig. (**2**). Estimated annual costs for human rights and community support for 2015, excluding nutrition are 1.5% (US $63.8 million) of the total projected spending on the ART programme. Human rights and community support costs are divided into those that are population-related (counsellors and training), site-related (information and monitoring), district-related (implementation, social mobilisation and supervision), and province-related (management, legal support and independent monitoring). Some costs will decrease over time. For example, it is assumed that the number of testing site locations will reduce from 2909 as the distribution of HIV prevalence falls over time. Costs relating to district and province do not vary and form 24% of costs at peak, but will rise proportionally over time as numbers tested and prevalence decrease. However, the costs of nutrition and adherence support are related to ART prevalence. Assuming treatment eligibility at CD4≤350, nutrition cost peaks at US $64.3 million annually.

## LIMITATIONS

Attempts to integrate human rights and community support into HIV programmes have often been limited to vague and often ineffective anti-stigma campaigns that fail to adequately address the broad range of human rights abuses. In some regards, our attempt to identify and cost public sector human rights and community support aspects of expanded ART programmes can be seen as replicating this overly narrow approach. Yet, while the interventions we have identified are limited to the immediate context of expanding ART, the identification and costing of these interventions can be seen as an important step to ensuring the expansion of ART programmes within a human rights-based approach. Costing categories are also important for policy discussions and decision-making when designing proposals and budgets for the expansion of HIV services. Engaging with stakeholders to build upon the current model to add additional services and costs, including broader “structural-rights interventions” [61], support for civil society and accountability measures will be important next steps in furthering models of expanded ART programmes. Although our costing categories and costs represent a best estimate from the literature, most of the interventions require evaluation and research to further develop the scientific evidence base regarding them. This is particularly relevant with regard to the most appropriate and effective interventions to address sociocultural barriers to HIV services. While addressing individual behaviours and bringing about cultural change is necessary, addressing the HIV epidemic is not possible without also addressing poverty, underdevelopment and illiteracy [61, 123]. Clearly, solutions to these issues involve long-term commitment at the international, national and community levels.

The costed interventions in this report seek to promote the goal of expansion of access to HIV prevention, treatment and care, as well as to provide community support and protect human rights. To be successful, it is essential that rights-based interventions are effective and have a strong basis in accountability and community engagement. Costed interventions could be implemented in collaboration with human rights and community support organisations that would optimally use the budget support to lead many of the activities. Support is also included for an independent auditing body that will provide feedback and a process for monitoring and reporting of human rights abuses. Collaboration and management of the resources in this sensitive area will not be easy and would require flexibility, openness and adaptability on all sides, as well as an ability to adjust and make improvements.

## CONCLUSION

There is increasing scientific evidence supporting the expansion of access to ART as part of HIV and TB prevention efforts. As part of the exploration of a theoretical expanded access to ART public health programme, we costed specific measures to ensure community support within a human rights-based approach. The programme as a whole would improve community access to health care, ensuring the basic human right to health. In 2015, the estimated human rights and community support programme elements totalled US $63.8 million or 1.5% of the projected annual budget and included training, education, supervision, monitoring and evaluation, transport and adherence support costing categories. A further US $64.3 million (1.5% of total costs) was added for nutritional support, recognising the negative impact of poor nutrition on health status. Although the total costs given are dependent upon the size and administrative structure of South Africa, many of the costing categories could be applied to other low- to middle-income settings with appropriate cost adjustments.

There are a number of significant challenges to implementing human rights and community support. Funding for public health is scarce and it is increasingly important for stakeholders to dialogue regarding the hard choices that are often necessary when deciding on the best use of limited resources. Community response and engagement in confronting HIV is critical, but communities are often faced with a number of challenges including lack of shelter, food and employment. These and other issues are likely to directly impact key programme and individual objectives such as accessing HIV services, retention, and adherence. Additionally, successfully including human rights and community support interventions likely requires that all parties work within a strong legal framework as well as an environment of trust, accountability and understanding—often the very elements that need to be addressed with human rights and community support interventions. Establishing a basic costed framework for discussion may represent an important step in successfully addressing human rights and community support.

Human rights in the context of HIV/AIDS has received considerable attention. Despite widespread recognition that both human rights and community support are essential, particularly for a successful response to HIV/AIDS, this paper represents one of the first attempts to translate this recognition into concrete costed interventions. Clearly further dialogue and careful consideration will be necessary to refine these potential interventions. Additionally, some of the interventions are already being used to improve services, and as more of these human rights and community support elements are incorporated into HIV programmes, the monitoring and evaluation of their impact should add to the scientific evidence base and policy discussions. The interventions included in the costing framework would represent a significant step forward in addressing both the HIV epidemic and ensuring a rights-based approach is used. However, responsibility for human rights and community support extends beyond the health sector, and further wide-reaching societal change is necessary to ensure that people living with HIV receive necessary community support and are free from human rights abuses. Although most communities have responded admirably to the significant challenge of HIV, there is disturbing evidence that fundamentally unsound interventions such as criminalisation and stigmatisation are gaining ground in some settings. The interventions suggested in this paper will likely be most successful when combined with social, cultural and political interventions to effect lasting and meaningful change, requiring commitment by national and international bodies.

## Figures and Tables

**Table 1.** Selected Non-Health Sector Human Rights and Community Support Interventions Not Included in the Costing Framework

**Legal reform** civil and criminal code law enforcement correctional system
** Changing cultural norms** equal rights for women and vulnerable populations preventing violence against women and children and other vulnerable populations engaging religious community engaging religious community for human rights and community support
** Addressing socioeconomic environment** poverty reduction economic development improve employment opportunities and conditions
** Improving educational system** primary, secondary and higher education health care providers pre-service training
** Multi-sector civil society support** support for civil society, human rights and community support organisations
** Strategic information and human rights monitoring** regular monitoring and evaluation dissemination of monitoring and evaluation results

**Table 2.** Summary of Measures to Protect Human Rights and Costs Involved

Measure	Level of Intervention	Human Rights Issue Targeted	Approximate Cost (US$)
**Educational programme linked to campaign**	National, community and individual	Stigma and discrimination Barriers to access	4.6 million *per annum*
**Human rights training for counsellors**	National training programme	Barriers to access including lack of education Consent, confidentiality and counselling Stigma and discrimination Identification and education of women suffering domestic violence Needs of marginalised populations Couples/serodiscordant couples specific training	12.6 million * per annum*
**Involvement of human rights organisations**	National	• Development of human rights tools (stigma scales, exit poll, training materials for counsellors, training materials for law enforcement bodies)	57,248 * per annum*
**Independent monitoring and evaluation contractor** • Collate and report on exit polls and feedback from site staff, counsellors and supervisors	National	• Accountability and feedback	150,000 * per annum*
**Legal assistance** Part-time lawyer (1 per province, 0.3 of a whole time equivalent) Full-time paralegal assistant §"Warm line" paging system One-time back-up fund for legal representation	Provincial	Discrimination against HIV positive individuals Consent, confidentiality and counselling	Lawyer (per province) US $7,524* per annum*, Paralegal assistant (per province) US $12,319. One-time back-up fund (national): US $100,000.
**Human rights provincial coordinators**	Provincial	• All human rights issues pertaining to expanded ART including provider-initiated testing and counseling (PITC)	111,353 * per annum*
**Shelters for women/ collaboration with existing shelters and support organisations**	District	• Violence against women	Uncosted
**Human rights district supervisor**	District	• All human rights issues pertaining to expanded antiretroviral therapy (ART) including PITC	652,899 * per annum*
**Monthly meetings with civil societies**	District	• Monitoring of human rights abuses	350,600 * per annum*
**Community support vehicle**	District	• Enable transport of human rights staff for monitoring	797,845
**'Know Your Rights' desk** Information Links to support services and emergency measures	Campaign site	Stigma and discrimination Violence against women	673,737 * per annum*
** PITC brochure costs**	PITC settings	Stigma and discrimination Violence against women	261,425 * per annum*
** Transportation costs**	Community/provincial	• Access to treatment	9.1 million
** Collaboration with prevention of mother-to-child transmission programmes and support groups for HIV-positive pregnant women or mothers**	Community and individual	• Discrimination against HIV-positive pregnant women/mothers	Cost included in comprehensive education and support package offered and in training of counsellors
** Additional measures to support most at risk populations**		• Stigma and discrimination	Counsellor training, part of broader campaign, no separate costing
**Education for women on domestic violence and available options**	National, community and individual	• Violence against women	Cost included in promotional and educational materials
**Human rights representative**	Site	• All human rights issues pertaining to expanded ART including PITC	Included in *Know Your Rights *desk cost
**Exit poll**	Site	• Accountability, feedback on satisfaction and human rights abuses	673,737 *per annum*
**Access to phone for emergency/helpline calls**	Community	• Violence and discrimination against HIV-positive individuals	Uncosted (as linked to counselling and relevant facilities)
** Community-based adherence support **	Community	• Support for HIV-positive individuals	Human rights/community support worker (per 120 HIV+ on treatment): USD $1440 *per annum* for 95% of 2.3 million people on treatment (total cost 26.1 million *per annum*) Human rights/community support supervisor (per community): USD $2667 *per annum* for 2909 communities (total 7.8 million *per annum*). Total $33,846,610
**Nutritional support BMI<18**	Community	• Support for HIV positive individuals	64.3 million (8.3% of those on ART)

ART, antiretroviral therapy; BMI, body mass index; MTCT, mother-to-child transmission; PITC, provider initiated testing and counselling: US$, United States dollars.

**Table 3.** Examples of Community Support and Human Rights Activities

**Raising awareness about HIV prevention, treatment and care and related stigma and discrimination issues at household and broader public levels** Home visits Giving talks at community forums (e.g. schools, municipal meetings, festivals) Disseminating information Hosting workshops for different target groups (e.g. children, adolescents, sex workers, pregnant women, drug users, women and girls, men who have sex with men) Liaising with key cultural organisations (e.g., religious community, army, political leadership)
**Strengthening community-driven referral system** Home visits Clinic attendance and liaison activities
**Community mobilisation** Treatment literacy efforts Setting up patient support groups Organising demonstrations Organising campaigns
**HIV prevention and treatment support** Male and female condom distribution Dissemination of prevention messaging Treatment literacy and adherence support Hygiene and public health messaging
**HIV care and support** Psychosocial support Home-based care Counselling Peer support

## ABBREVIATIONS

AIDS: = Acquired Immune Deficiency Syndrome
ARASA: = AIDS and Rights Alliance for Southern Africa
ART: = Antiretroviral Therapy
BMI: = Body Mass Index
DC: = District of Columbia
DoH: = Department of Health
DOT: = Directly Observed Therapy
HAART: = Highly Active Antiretroviral Therapy
HIV: = Human Immunodeficiency Virus
HR: = Human Rights
IMF: = International Monetary Fund
MDGs: = Millennium Development Goals
M&E: = Monitoring and Evaluation
MTCT: = Mother-to-child Transmission
PITC: = Provider-Initiated Testing and Counselling
TB: = Tuberculosis
UK: = United Kingdom
UNAIDS: = Joint United Nations Programme on HIV/AIDS
US(A): = United States (of America)
VCT: = Voluntary Counselling and Testing
WHO: = World Health Organization
